# Feasibility, useability and acceptability of technology-based interventions for informal cancer carers: a systematic review

**DOI:** 10.1186/s12885-018-4160-9

**Published:** 2018-03-02

**Authors:** Natalie Heynsbergh, Leila Heckel, Mari Botti, Patricia M. Livingston

**Affiliations:** 1 Geelong, Faculty of Health, School of Nursing and Midwifery, Deakin University, Geelong, Victoria 3220 Australia; 2 Geelong, Faculty of Health, School of Nursing and Midwifery and Epworth HealthCare, Deakin University, Geelong, Victoria 3220 Australia; 3 Geelong, Faculty of Health, Deakin University, Geelong, Victoria 3220 Australia

**Keywords:** Cancer, Carer, Technology, Web, Smartphone applications, Review

## Abstract

**Background:**

Carers looking after someone with cancer often experience negative impacts on their own health. M-health interventions have been designed to provide information and support to patients and their carers. However, the effectiveness of technology-based interventions for carers is less well understood. The objectives were to assess the feasibility, useability and acceptability of technology-based interventions among carers of people living with cancer.

**Methods:**

A systematic search of the CINAHL, MEDLINE and PSYCINFO databases was performed using terms related to web-based interventions and smartphone applications, carers and cancer. Studies were included if a randomised controlled trial or pilot study was conducted, focused on adult carers looking after another adult with cancer and were published between January 2007-June 2017. Articles were excluded if they reported qualitative results only or were evaluating existing websites and applications. Feasibility was measured by attrition, recruitment rates and frequency of intervention use; useability was measured by the ease of intervention use and the role of features to minimise errors in use. Acceptability was measured by carers’ perception of the appropriateness of the content and their ability to incorporate the intervention into their daily routines.

**Results:**

Of the 729 articles, six articles met the inclusion criteria. Attrition ranged from 14% - 77%, recruitment rates from 20% - 66% and intervention useability varied across studies. Half of the studies implemented measures to improve useability. Overall, carers rated the content of the interventions as appropriate and reported improved knowledge and communication. Acceptability was further demonstrated as carers preferred the flexibility available with web-based interventions.

**Conclusions:**

Technology-based interventions are suitable for use among carers of people with cancer. Further research is required to fully assess the impact of technology as an information and support mechanism for carers.

## Background

Cancer is a significant public health issue in Australia; it is estimated that 134,177 people will be diagnosed with cancer in 2017 [1]. Improvement in treatment and early detection has changed the classification of cancer to a chronic illness [2].

Financial limitations on health care systems has seen a push for patients to be discharged from hospital earlier, meaning that those with chronic illnesses are caring for their disease in the community [3]. It is common for a person with chronic illness to have someone who provides care to them at home on an ongoing basis [3]. Carers are most typically patients’ spouses, parents, adult children or other relatives [4]. These carers are known as ‘informal carers as they are not paid for the care that they provide and often lack the knowledge, skills and resources necessary to provide holistic care for the patient [5]. In 2015, there were 2.86 million informal carers in Australia [6]. Informal carers can provide up to 40 h a week of unpaid care [7], saving communities approximately $60.3 billion in healthcare costs in 2015 [6]. Carers can experience negative impacts on their own physical, mental and social wellbeing and financial status during their caring role [8–10].

In Australia there are limited supportive measures available to carers of people with cancer [11, 12]. With their growing popularity and functionality, technology based tools may allow individuals to be proactive about their health, to create social networks and to have access to information specific to their needs [13, 14]. For carers, particularly those who are time poor or are unable to leave the person with the illness unattended, technology based interventions have the ability to be accessed in the privacy and comfort of one’s own home, at a time convenient for the individual [15]. Overall, 92% of the Australian population access the Internet either through a computer or smartphone/tablet device [16]; this presents novel opportunities for the provision of information and support to large audiences. E-health interventions have been shown to have positive impacts on carers’ psychological health, knowledge and skills, social support, communication and quality of life across a variety of illnesses and among carers looking after children with illness [17].

Previous reviews have focused on the efficacy of e-health interventions for patients and carers of people with cancer. Badr et al. [18] assessed how web-based programs can improve health communication in carer and patient dyads and Slev et al. [19] performed a meta-analysis of reviews of e-health interventions among carers and patients with cancer. Findings indicated that no studies reported on the effects of e-health on carer outcomes specifically. Of the articles included in the review one focused on smartphone applications (apps) [20]. This review analysed the type of apps that were available for purchase through app stores, rather than trials of apps among carers [20]. Four of the studies included in this review also featured in a previous systematic review by Kaltenbaugh et al. [21] who assessed the impact of web-based interventions on physical, social, psychological, and financial outcomes of carers.

In developing a technology-based intervention, it is important to determine if the content of the intervention is useful and acceptable for end users, if the intervention can be used to sustain their wellbeing and if technology itself does not create any barrier to access [22]. Demographic characteristics such as age, gender and income may have an impact on the success of technology-based interventions. Age may have an impact on the likelihood of carers using technology over other information methods and possessing the skills to navigate technology-based interventions [23]. Further, needs can differ across genders [24, 25] and it is important to assess whether interventions are successful at addressing the needs of all users. While technology-based interventions have the capacity to reach larger numbers of people compared to face-to-face interventions, there is still the potential for bias to occur, as people on higher incomes are more likely to own computers and smartphone devices or have continuous data roaming capabilities than those with lower incomes [14]. These are important factors when trialling technology based interventions.

The purpose of this systematic review was to assess the feasibility, useability and acceptability of technology-based interventions among carers of people with cancer. Feasibility was measured by attrition, recruitment rates and frequency of intervention use [22]; useability was defined as features of technology that helped to minimize confusion and errors in the program’s use [26] and acceptability was the observed appropriateness of the intervention [22] and the ability of participants to incorporate the intervention into their daily routines for sustained use.

## Methods

### Search methods

Three databases were systematically searched for published articles between January 2007–June 2017: CINAHL, Medline and PsycInfo. Abstracts were downloaded into Endnote X7 and all duplicates were removed. Additional articles were obtained from the reference lists of articles accepted into the review.

### Search terms used

Search terms included: cancer OR neoplasm* OR tumour OR tumor OR oncol* OR malign* OR metast* AND carer* OR caregiver* OR spouse* AND Smartphone* OR “smart phone*” OR mobile OR “cell* phone*” OR online OR web OR computer* OR PC. The search strategy allowed for the inclusion of MesH terms “caregivers”, “smartphone”, “neoplasms”, “computers” and “cell phones” and CINAHL headings “cellular phone” to systematically search databases.

### Selection of studies and data extraction

Titles, abstracts and full text were initially screened by one author (NH) and relevant articles were subsequently reviewed by LH, MB and PL. Data were extracted by NH, by analysing the outcomes of each study against the measures of the current review. Discrepancies were discussed by all authors until a general consensus was reached.

The methodology of each article was assessed for risk of bias against the Cochrane Handbook for Systematic Reviews of Interventions, including selection, performance, attrition, detection and reporting bias [27].

### Inclusion criteria

Studies were included if they were published in peer-reviewed journals in the period of January 2007 to June 2017 and reported on the feasibility, useability or acceptability of web-based and smartphone application interventions. Articles were considered if they:

- included adult informal carers of other adults diagnosed with cancer
- included cancer dyads, however carer outcomes needed to be reported separately
- comprised randomised controlled trials (RCT), pilot studies or single arm case studies

### Exclusion criteria

Studies were excluded if only qualitative data were provided, if pre-existing websites and applications were assessed and if they were published in languages other than English.

Three articles were protocol papers [28–30], three were systematic reviews [18, 19, 21], two provided qualitative findings only [31, 32], one provided mixed methods results but only distinguished between carer and patient results qualitatively [33], one provided user feedback during web development [34] and one was a published abstract from a conference presentation [35]. These articles were excluded during title, abstract and full read screening and were not included in the review.

### Outcomes measures

There have been inconsistencies in the way that feasibility has been measured in the past. Measures of feasibility have variously included: recruitment rates, attrition, intervention use including number of sessions started, number of sessions completed and useage statistics, effect sizes and post intervention follow-up including questionnaires and qualitative interviews [18, 36–40]. The most common measurements of feasibility were recruitment rates, attrition rate, and the frequency of use of the intervention. Accordingly, these measurements were used to determine overall feasibility across the studies included in this review.

Useability was assessed by participants’ ability to successfully use interventions, as well as specific design features to minimize confusion and errors in the program’s use.

Acceptability was measured by carers’ perceptions of the appropriateness of the content and their ability to incorporate the intervention into their daily routines.

Secondary information also recorded included carers’ age, gender, annual income, and the country in which studies were conducted. Information about carer age, gender and income allowed for discussion about the impact of participants’ demographic characteristics on technology use and potential limitations of the feasibility, useability and acceptability of interventions. Identification of the country where the studies were conducted allowed for an assessment of the current scope of research internationally.

## Results

**Fig. 1:**
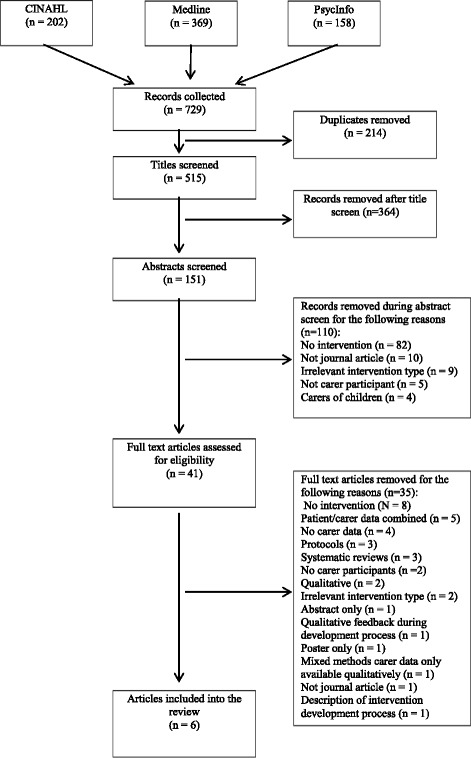
PRISMA flow diagram of literature search

Across the three databases, 729 articles were retrieved: CINAHL = 202, Medline = 369 and PsycInfo = 158, from which 214 duplicates were removed. Remaining articles were then screened by title and abstract for eligibility, and a further 474 articles were removed due to ineligibility, three were protocols, three were systematic reviews and one was a conference abstract. The full text was retrieved for 41 articles, and these were again screened against the inclusion criteria. A further 28 articles did not meet the criteria and were removed. Additional articles were retrieved from the relevant systematic reviews and screened for inclusion, no new articles were obtained by this method. Finally, six manuscripts were included in this review. This study followed the Preferred Reporting Items for Systematic Reviews and Meta-Analysis (PRISMA) structure for searching and screening articles to be included in the review [41]. Figure 1 provides a PRISMA diagram of the search strategy. Table 1 outlines the key information of the included web-based interventions.

**Table 1:** Findings for the feasibility, useability and acceptability of technology-based interventions for cancer carers

	Sample characteristics					Methodology			Results				
Reference	Cancer type	Country	N=	Mean age	Female (%)	Design	Study arms	Intervention	Study primary outcomes	Feasibility	Useability	Acceptability	Limitations
Chih, DuBenske [42]	Advanced: breast, prostate and lung	USA	217	56	64.2	• RCT • 6 and 12-month follow-ups	1) CHESS 2) CHESS with clinician report (CR). CR sends reports to the clinician for timely management of symptoms	• Both groups completed a check-in every seven days and provided information about their needs and patients’ symptoms. • Both groups had access to CHESS: information, communication and training services • CR created a report that was made available to clinicians to review patient symptoms	1) Caregiver preparedness No significant effect. 2) Physical burden No significant effect 3) Negative mood Carers in the CHESS with CR group had less negative mood than those in the CHESS only group at 6 months (*p* = 0.009) and at 12 months (*p* = 0.004).	• Recruitment rate 25% • Attrition rate 39% at 6 months. • Attrition rate 52% at 12 months. • 26% of people in CHESS + CR and 21% of people in CHESS did not use Check in	• Telephone or face-to-face training • Distribution of computer and system manuals • Available technical support line	–	• CR alone could not be assessed because it was integrated in CHESS. • Carers with fewer needs may have been more likely to complete the 12-month follow-up. • The sample was most well educated, Caucasians.
DuBenske, Gustafson [43]	Advanced lung	USA	246	55	68.3	• RCT • 24 -month intervention • 2 monthly follow-ups. • 6-month data reported on	1) CHESS + CR 2) CHESS 3) Internet access with a list of recommended websites	• Participants in the CHESS groups had access to information, communication and training. • Participants were encouraged to log in weekly, but natural useage was observed.	1) Disruptiveness No significant effect. 2) Burden At 6 months carers in the CHESS + CR group had lower burden than carers in the Internet only group (*P* = 0.021). 3) Negative mood At 6 months carers in the CHESS + CR group had lower negative mood than carers in the Internet only group (*p* = 0.006).	• Recruitment rate 62% • Attrition rate 31% at 6 months. • Nearly 50% did not return 6-month follow-up surveys • 73% of users logged into CHESS at least once • Carers logged in on average 14.6 times, viewed 293 pages and used the system for 50 min each month • 27% of users in Group 1 used the system minimally or not at all	• Computers and laptops provided • Internet use paid for • Distribution of computer and user manuals • Telephone training offered for all participants, 59% completed training	–	• Low recruitment and the removal of the CHESS only arm (Group 2). • Providing carers with Internet access may have been sufficient to address some carers’ needs, decreasing any effects of CHESS.
Namkoong, DuBenske [44]	Advanced lung	USA	285	55	68%	• RCT • 24-month intervention • 2-monthly follow-ups • 6-month data reported on	1) CHESS 2) Internet only	• Carers in the CHESS group had access to information, communication and training services. • Participants were encouraged to log in regularly, but natural useage was observed.	1) Caregiver bonding At 6 months a positive effect (*p* = 0.05) was seen in carers bonding with one-another in the CHESS group compared to the Internet only group. 2) Association between CHESS use and coping strategies No significant effect 3) Association between caregiver bonding and coping strategies. Positively associated with active behaviour (p = 0.05), positive reframing (p = 0.05) and instrumental support (*P* = 0.01)	• Recruitment rate 43% • Attrition rate 63.5% • Only 104 carers completed to 6 month follow up	–	–	–
Northouse, Schafenacker [36]	Advanced lung, colorectal, breast and prostate	USA	44	50	60.5%	• Single arm feasibility study • 6-week intervention • Pre and Post surveys		• Carer/patient dyads completed three modules each two weeks apart. • Intervention content was tailored depending on information provided in baseline questionnaires. • Content included the impact on cancer on the family, addressing concerns and finding support and future planning.	1) Emotional distress Small reduction in distress (d = 0.28). 2) QOL Small improvement in QOL (d = 0.20). 3) Social support Small improvement in social support (d = 0.33). 4) Self-efficacy Small improvement in self-efficacy (d = 0.40).	• Recruitment rate 51% • Attrition rate 14%	• A help button was available for carers if they require assistance. Few carers utilised this, and only when they required help with passwords • The program was reported highly as easy to use	• The intervention was accepted as it could be completed at home and at a time convenient to the participants • Some participants requested more information about symptom management and coping	• Unable to assess the efficacy of the intervention as there was no control group
Scott and Beatty [37]	Breast, colorectal, prostate, thyroid, ovarian, testicular and angio-sarcoma.	Australia	13	48	67%	• Case study • 6 module cognitive behavioural therapy course • Baseline, 1-week post intervention and 3 months post intervention follow-up		• A cognitive behaviour therapy programme including education, worksheets and survivor stories. • Modules released each week. • Program designed for patients, carers asked to take patient perspective for aspects that did not directly apply to them.	1) Negative affect Large reduction in negative affect (d = 0.88) 2) Cancer specific distress Small reduction in distress (d = 0.37). 3) OQL role functioning Small increase in role functioning (d = 0.34). 4) QOL emotional functioning Moderate increase in emotional functioning (d = 0.62).	• Recruitment rate 20% • 31% attrition to the end of the intervention • 77% attrition to the follow-up questionnaire • Five participants completed the program • Four participants completed 2-5 modules • Four participants completed one or none of the program	–	• 78% stated the program was helpful • 33% stated the intervention needed a stronger focus on carers • Qualitative findings showed mixed results for the acceptability of the intervention, some preferring the convenience of web-based interventions and some finding them impersonal	• Dropouts were more likely to have better psychological outcomes at baseline • Carers who completed the intervention were more likely to be in the post-treatment phase • No control group
Song, Rini [38]	Prostate	USA	25	59	100%	• Feasibility study. • Eight-week intervention • Pre and post surveys • Qualitative post-intervention assessments		• Dyads completed seven education modules: two mandatory and five optional. • Modules included information, links to videos for skills training and assignments. • Each module took 10-20 min to complete. • Assignments required extra time to complete • Dyads had access to resource centre with online resources and links to organisations.	1) QOL Small increase in social quality of life (d = 0.25). 2) Couple communication No effect 3) Perception of patients’ symptoms Small increase in effect size from d = 0.18 pre intervention to d = 0.51 post intervention.	• Recruitment rate 51% • Attrition rate 15% • On average carers spent 43.99 min on the intervention (patients’ 41.99 min)	• The intervention was rated as easy to use, concise, easy to understand and engaging with different formats of information (text and videos) • There were no issues with Internet connectivity	• The intervention was accepted for improving knowledge, symptom management and communication Carers valued that they were able to tailor the modules to their own needs	• Inclusion of patients and carers with relatively low symptoms/ needs (19 months post diagnosis) • Exclusion of non-partnered patients/carers

Note: A dash has been used in cells where information was not reported on. CR refers to clinician report and QOL refers to quality of life

p = statistical significance < 0.05

d represents effect sizes where 0.2 is small, 0.5 moderate and 0.8 large effect

### Design and format of interventions

Of the six studies reviewed, three studies used a RCT design [42–44], two were single arm feasibility studies [36, 38] and one a case study [37].

Five studies were undertaken in the USA and recruited dyads of carers and patients [36, 38, 42–44] and one study was undertaken in Australia and recruited carers only [37].

In two studies, both carers and patient participants had access to the intervention [36, 38]. In three studies, information about patients was collected but patients did not use the interventions tested [42–44]. The carer-only study included participants whose significant others’ had the option to participate in a patient version of the intervention [37].

Scott and Beatty [37] (*N* = 13) conducted a web-based six-module course with modules released sequentially each week. Follow up assessments occurred one week and three months after commencement of the course. Song et al. [38] (*N* = 22) conducted a seven-module course, with each module lasting for 10-20 min, with additional time required for assignments. Two modules were mandatory and five were optional. Modules could be completed weekly, or within the couples’ preferred time frame. Follow up assessments occurred between weeks three and eight, depending on how many modules were completed [38]. Northouse et al. [36] (*n* = 38) provided a six-week intervention with fortnightly modules and carers completed a follow up assessment during week eight. Three studies used a similar online system called Comprehensive Health Enhancement Support System (CHESS) where carers in each of these studies accessed the intervention for two years and completed follow-up assessments every two months [42–44]. In the trial conducted by Namkoong and colleagues [44] (*N* = 285) and DuBenske et al. [43] (*n* = 246) carers were encouraged to log in regularly, however they could use the intervention at their own leisure to allow observation of natural useage. In the CHESS trial by Chih et al. [42] (*N* = 217) carers were encouraged to log in every week to complete “Check-in” making notes of carers’ needs and patients’ symptoms.

### Content of interventions

Three studies used the framework called CHESS [42–44]. CHESS is an interactive online cancer communication system that enables carers and patients to monitor patients’ symptoms. CHESS includes several components including symptom tracking, communication with others in a similar situation, coping skills training and access to information resources. Namkoong et al. [44] adapted CHESS to focus on patients with advanced lung cancer, included carer participants and an Internet only control group. In later trials CHESS incorporated a Clinician Report (CR), which included sending the treating doctor a report of the patients’ symptoms for immediate review or discussion at the patients’ next appointment, depending on the severity of the symptoms reported [42, 43]. Northouse et al. [36] adapted a face-to-face program called FOCUS into a web-based trial to test the concept of family involvement. Dyads completed the intervention together every fortnight and received tailored messages containing the intervention content designed to improve communication, support and disease management. Song et al. [38] trialled a couples-based intervention to manage symptoms and improve quality of life (QOL) of patients and their carers. The two mandatory topics included communication with the healthcare team and survivorship issues such as distress and relaxation. The five other modules included management of specific symptoms such as bowel and urinary issues and could be explored depending on carers’ and patients’ needs. The intervention included: information, skills training and couple-based assignments [38]. Scott and Beatty [37] conducted a feasibility study of an online program that was originally designed for the person living with cancer, to test its applicability to carers. The six-weekly module program covered topics such as beginning treatment, coping with symptoms and emotional distress, body image, communicating with family and friends and completing treatment. The intervention included psycho-education, cognitive behaviour worksheets and survivor stories [45].

### Participant demographic characteristics

Across studies, female carers accounted for more than 60% of participants and carers’ age ranged between 48 and 59 years. Annual income was measured differently within each study. Two studies reported only carers’ incomes. The majority of carers in the study by Scott and Beatty [37] had an annual income of > AUD$35,001 (54%) with 46% earning < AUD$35,000. Northouse et al. [36] measured carer income from US$15,000-$50,000, US$50,001-100,000 and > US$100,001 (15.8%, 36.8% and 31.6%, respectively). Two studies reported on annual household income. Song et al. [38] measured income between < US$30,000, US$30,000-$75,000, > US$75,000 (18%, 41% and 41%, respectively). Chih et al. [42] measured income from < US$40,000, US$40,001-$80,000 and > US$80,001 (32%, 34% and 25%, respectively). Two studies did not report on income [43, 44].

Types of cancers included breast, prostate, lung, colorectal, thyroid, ovarian, testicular and adenocarcinoma. The majority of studies focused on carers of patients with advanced cancers [36, 42–44].

### Feasibility

Recruitment rates varied between 20% and 66%. Due to low recruitment, a study arm had to be removed from the design of two studies [42, 43]. Attrition rates varied between 14% and 77%. In three studies, the reported use of the intervention for over 25% of their participants was minimal or not used at all [37, 42, 43]. Two studies collected useage data, with one study reporting an average usage of 50 min per month for six months [43] and the other of 44 min over the duration of an eight-week intervention [38].

### Useability

In three studies enablers were provided to improve the useability of the interventions. This included providing participants with computer and internet access [43], having a help button installed in the program [36], providing training in using the program [42, 43], distributing written instructions [42, 43], and having contact details to speak to someone when experiencing difficulties [42]. Three studies did not provide any useability features to improve ease of intervention use [37, 38, 44] and two did not report on useability outcomes [37, 44]. An issue of useability noted was forgetting passwords to login to the program [36].

### Acceptability

Perceived benefits of the web-based interventions included being able to complete the intervention at home, at the carers’ own time preference [36] and in one study, being able to tailor the intervention to the individuals’ needs [38]. Participants found the interventions helpful in improving their knowledge and communication [38]. Carers perceived the provision of more specified information on symptom management and coping as beneficial [36]. One study reported conflicting results about the acceptance of web-based interventions, some carers preferred the flexibility while other carers rated the intervention as impersonal and wanted a combination of face-to-face contact and web-based support [37]. Half of the studies did not report on acceptability outcomes [42–44].

## Discussion

This review assessed the feasibility, useability and acceptability of technology-based interventions for carers of people with cancer. Six studies using web-based interventions were included in the review; the search did not return any studies using smartphone applications.

Overall, findings were generally positive for the feasibility of web-based interventions with a range of recruitment and attrition rates. For example of the four studies focusing on carers of patients with advanced cancers [36, 42–44] two had attrition rates greater than 50%, as carers were likely to drop out after the death of the patient [42, 44]. However, these higher attrition rates are comparable to other non web-based intervention studies involving carers of, and patients with, advanced cancers and do not suggest that stage of cancer has an impact on the feasibility [43, 46]. Intervention content may have impacted on feasibility. Scott and Beatty [37] showed that a programme designed for patient use had 31% attrition at the end of the 6-week programme and one third of participants stated that the content was not focused enough on carers. Similarly, intervention length may have impacted on attrition as three studies had follow up periods of 6 months or greater and reported attrition rates between 31 and 63.5% [42–44], however as these studies also included carers of people with advanced cancers, it is possible that the duration of the interventions combined with stage of cancer caused large attrition rates. Studies with intervention durations between six to eight weeks and whose contents focused directly on the needs of carers had smaller attrition rates of 14 to 15% and may be more feasible [36, 38]. More research is required to assess the optimal length of interventions in combination with stage of cancer to enhance carers’ capacity to engage with interventions.

Reasons for poor recruitment rates tended to be methodological where follow-up contact with potential participants was not part of the procedures, and/or of a personal nature where carers were too busy to participate, too unwell, were coping well or where patients did not nominate a carer to participate [36, 37, 46]. Three studies highlighted technology-specific barriers to recruitment, with carers either lacking the skills necessary to use web-based interventions [37, 44] or preferring programs delivered in person as recruitment was lower for the web-based intervention than in a previous study using the same intervention in face-to-face settings [36]. This suggests that carers’ apprehension in using technology, or their ownership of technological devices may have been a barrier to their participation [36, 37, 44, 46]. However, as familiarity with the use of technology increases, the ability for people to receive the benefits of technology-based interventions can be maximised.

Useability was demonstrated in half of the studies by inclusion of instructional information and technical support. Orientating participants to the intervention may represent a useful approach to helping them better understand the content of the intervention and increase usage. In two of the studies [42, 43], which provided computers, paid Internet access and useability enhancements in the form of training sessions, useability did not appear to have an impact on the feasibility or acceptability of the interventions. Despite carers having fewer financial barriers and the availability of useability enhancements, recruitment rates were between 25 and 62% and attrition between 31 and 52%. These studies focused on carers of people with advanced cancers and had long intervention periods, which may have impacted on recruitment and attrition. The third study [36], which implemented useability enhancements in the form of a help button had a recruitment rate of 51% and attrition of 14%, however few carers used the help button. This support tool was therefore less likely to impact on the feasibility of the intervention and lower attrition may have been in part, due to the shorter intervention period of six weeks.

Acceptability measures highlighted that carers liked the flexibility provided by web-based interventions as they could complete them at home and in their own time. The interventions improved carers’ knowledge, but some carers reported a need for a higher level of carer related information, as the information provided was not specific to their needs. Carers overall liked web-based interventions, however some carers preferred face-to-face communication. Only three of the studies reported findings relating to the acceptability of interventions, therefore it is not possible to generalise these findings to all web-based interventions.

In the study reports, discussions of limitations highlighted that completion rates could be related to patients’ stage of illness and carers’ level of need and burden [37, 42, 44]. Carers who experienced higher levels of unmet needs and burden may not have completed interventions, potentially leading to biased results about the efficacy and applicability of interventions to carers of people with cancer [37, 42]. The study design and intervention design limited studies’ ability to properly measure and generalise the effects of the interventions [36–38, 42, 43]. Annual income did not present as a barrier to intervention participation [36–38, 42], however, it is possible that carers who did not own technological devices due to low income did not have the opportunity to participate in the studies. Further, the distribution of age and gender was similar across all studies and did not appear to present a barrier to use, however it is possible that interventions would not have been as well received among carers of different ages or different genders including males, transgender people and people with gender diversity. More research is needed to assess the appropriateness of technology-based interventions for people with varying ages, genders and incomes.

This review should be interpreted with caution as it included studies with mixed methodologies between study designs, intervention lengths and intervention content. Intervention length differed with some studies reporting on outcomes after eight weeks, and others after six-months. The content of interventions varied in focus between cognitive-behavioural therapy and information and communication with healthcare teams. Because of the differences in methodologies it is difficult to draw direct comparisons between studies and provide a clear finding of the feasibility, useability and acceptability of interventions as a whole. Recruitment rates across the studies were low and, while consistent with previous research involving cancer carers and patients this may have resulted in selection bias. Reasons for low recruitment already discussed included methodological, personal and technology factors. Three studies may have been subject to further selection bias due to their use of single arm study designs [36–38]. Further varied and high attrition rates may have resulted in attrition bias, as outcome data were incomplete across studies. As a result it is possible that this review has provided overly positive results for web-based interventions, as it is possible carers who participated were less burdened and more likely to participate in a research project. The lack of studies involving smartphone applications limits the interpretation of feasibility, useability and acceptability of technology-based interventions.

Quality assessments are used in systematic reviews to assess the internal validity and quality of reporting in each study [47, 48]. A quality assessment was performed on the studies using an RCT design, each study was scored as being of fair quality as there was some potential for bias to occur due to: attrition rates in excess of 20% [42–44], failure to blind participants and clinicians [42–44] and to report the randomisation process [44]. A quality assessment of studies using other design methods was not performed. The purpose of this review was to assess feasibility, useability and acceptability of technology-based interventions. Attrition and varied study designs with the inclusion of qualitative data allowed for in depth analysis of factors contributing to technology use. As few articles met the inclusion criteria, exclusion based on quality would have further limited the results of this review.

The findings of this review suggest that web-based interventions may have a positive impact on carers’ social and psychological wellbeing and are consistent with previous research assessing a variety of e-health interventions [17]. Understanding factors that may contribute to the success and sustainability of web-based interventions among carers of people with cancer is essential.

These findings inform future carer-specific web-based interventions, as the elements of feasibility, useability and acceptability identified can be incorporated in their design and implementation. That the majority of studies included in this review were conducted in the USA shows a need for further research in the area of carers of people with cancer globally. Smartphone applications present a new way of reaching the population to deliver information and support. The lack of previous research warrants future investigations.

## Conclusion

Technology has the potential to provide unprecedented support to carers of people living with cancer. Limited evidence suggests that web-based interventions are feasible, useable and acceptable among carers. Further research in this area is required to fully address the potential impact of technology in supporting carers of people with cancer. Findings from this review may be adapted to other technology formats including smartphone applications.

## Abbreviations

App: Application
CR: Clinician report
PRISMA: Preferred Reporting Items for Systematic Reviews and Meta-Analysis
QOL: Quality of life
RCT: Randomised controlled trials
